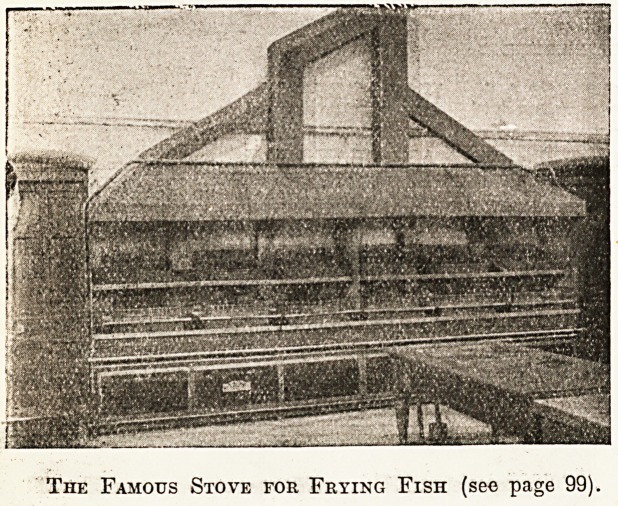# The Mental Hospital Kitchen

**Published:** 1912-04-27

**Authors:** 


### THE MENTAL HOSPITAL KITCHEN.

DR. ROBERT JONES ON THE KITCHEN AT CLAYBURY.

The size of Claybury Mental Hospital, covering over twenty acres of ground, the number of its patients, which is nearly 2,500, and the very large staff, about 400 in number, which is necessary to run the institution, would alone make the kitchen here of extreme interest. But it was upon the more specialised subject of the place of the kitchen in a modern mental hospital, from the medical point of view, that Dr. Robert Jones was good enough to discuss with our Commissioner the other day.

### An Evidence of Kitchen Care.

"You have only," he said, "to glance at the curriculum which the nurses who are trained here have to undergo to see what an important part is played, firstly, by the diet, and, secondly, by the methods of preparing and of serving food in the modern treatment of mental cases. In the first place, I would remind you that for almost every fresh case which is admitted here a special diet is prescribed, and last year there were over 400 new admissions. Experience of administration," he went on, as we made our way into the specious kitchen, "has convinced me that it is not sufficient to prescribe a merely nutritive diet, but it must also be attractive. The class from which our patients come has actually conditioned the contrivances of our kitchen, sometimes at considerable cost. For instance, look at this great stove (page 100), which is entirely used for cooking fish, and is in its way the prototype of many similar stoves which have now been adopted in other mental hospitals of the country. Its secret and its originality consist in devising a means for frying all fish in dripping, in the same fashion as the patients have for the most part seen it served previously in their own homes. These great pans are filled with dripping, and by an ingenious contrivance this dripping is continually returned as each wire dish is emptied of its grilled contents and the dripping in its fluid state is conveyed to the rear of the pan by the collecting pipe which you can see overhead. There is no doubt that this elaborate system of frying fish, which many people have come to see, not merely adds to the flavour of the fish, but saves waste, and has increased the popularity of this part of the diet among the patients."

Passing on further we then came to what looked like an enormous cage for rats! "This," said Dr. Jones, "is our tea-infuser. It has an interest, in that we found in practice, as the steward will tell you, that the ordinary plan of taking out the infuser after the tea had been allowed to draw was a wasteful one, because inspection showed that an important amount of tea in the centre of the infuser was never 'made' at all, and was more or less in the same condition as that in which it had been before it was unpacked. We have now a device for keeping the infuser in motion, so that the essence of all the tea may be used."

A View of the Claybury Kitchen.

The Serving Problem.

As we were passing, the trolley carriages with the patients' dinners were being wheeled into the corridors.

"How about the serving of your dinners?"

"As Claybury, which has been built for nearly twenty years, is famous for its corridors, the problem of serving the dinners has been a very great one. This dinner here, perhaps, may have to be wheeled a quarter of a mile away. In this trolley, therefore, you will find every division jacketed with hot water and so covered that it is practically impossible for any of the heat to be lost."

"I notice that gas ovens are used."

"Yes, and they are very large ones; a man can almost stand up in them. Indeed, one virtually does so every day when all their racks are removed, so that the inside of the oven can be completely cleaned and scoured. Beef-tea here is also an important article of diet. We have it in two grades, and it is one of the things with which special pains are taken to teach all the women members of the staff who are trained here. They have a special course in it, as well as in the making of jellies and custards."

Dr. Jones then led the way into the bakehouse, where the eight-pound loaves were being made. "These cakes," he

The Famous Stove for Frying Fish (see page 99).

said, "remind one of one's schooldays, the seed-cake and the plum cake being both very popular. There is here, too, a special contrivance for the cutting of hot-cross buns for Good Friday, which is only used at one season of the year. When in use it cuts thirty-seven hot-cross buns at one time by an ingenious mechanism that requires some understanding. As regards the bakehouse ovens, the fires are lit on Saturday, let out on Sunday, but they preserve sufficient heat to do all the baking on Monday morning."

The Superintendent's Point of View.

"Now, apart from some of the more typical contrivances, which I have shown you, the mental hospital kitchen, from my point of view, as medical superintendent, does not end here. You may have noticed that standing by the trolleys as they were going out there was a lady inspecting them. The apparatus is no use without the personal supervision, and my aim as superintendent must be to see that the diet and the food are carefully checked and properly carried out. Ever since the treatment of mental disease began, in the modern sense, by recognising the patients as human beings, it has been the increasing endeavour to make them as well and healthy and also as cheerful and bright as possible. Now, all that is impossible if the details of their meals, which, naturally, are even more critically regarded by many of them than by normal persons, are not very carefully arranged. The result is that every care is taken to ensure attractiveness and variety of diet. And in dealing with this class of patient food has a special importance, because the general health of many here is not good, and, consequently, in ordering diets one is not always bound down to the narrow list which is imposed on hospital patients whose maladies are only physical, and not both physical and mental as they are here. The kitchen, therefore, has two sides—the mechanical side, which has its own interests, and what may be called the psychological side, where prejudices have to be studied from every standpoint.

"All the very elaborate arrangements that have to be made for the population of a small city such as is lodged here are subsidiary, yet important as leading up to this. For once the old attitude represented by the custom of shackles was done away with, it was clear that the kitchen offered one of the readiest ways of improving the outlook and the well-being of the mentally afflicted. The introduction of gas cookery and the infinite number of gas rings in the kitchen at Claybury have thus a meaning which is not at first fully apparent. All these appliances have their value in coming to the assistance of treatment and in ensuring that the serving of the food, which, as I said, the size and planning of our buildings have made a difficult problem, should be accomplished with that nicety that the modern treatment of mental cases insists on in its fulness."

At this point Dr. Jones opened a door of one of the workrooms, from which a voluntary party of patients, who had been making almost all the linen that has been required at the last new asylum for London near Epsom, had just left for lunch.

"Are they rewarded for their work?"

"Yes," by privileges of diet: jam, extra cake, tea and sugar, cocoa, biscuits, or whatever dainty they prefer is given to them by way of encouragement. This is a simple instance of the importance we have to attach, and of the value which the patients attach, to their meals. Physical contentment in its widest sense has to be aimed at, for just as excitement will calm down and be restored to quietude by the anabolism which nourishing diet favours, so this in proportional degree is equally true of the less acute forms of insanity. This is why the kitchen should be one of the things best worth seeing in the mental hospital of to-day."

## Figures and Tables

**Figure f1:**
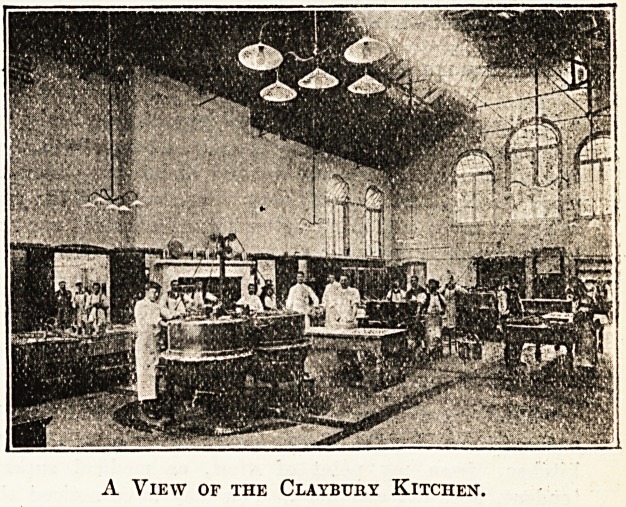


**Figure f2:**